# Genome Sequence of Aeribacillus pallidus W-12, a Thermophilic Bacterium Containing Possible Biodesulfurization and Biodenitrification Pathways

**DOI:** 10.1128/MRA.01570-18

**Published:** 2019-04-18

**Authors:** Chenchen Peng, Bingling Zhang, Wei Wang

**Affiliations:** a Key Laboratory of Molecular Microbiology and Technology, Ministry of Education, TEDA Institute of Biological Sciences and Biotechnology, Nankai University, Tianjin, China; b Tianjin Key Laboratory of Microbial Functional Genomics, TEDA, Tianjin, China; University of Maryland School of Medicine

## Abstract

Aeribacillus pallidus W-12 is a thermophilic bacterium isolated from Dagang oil reservoir sewage in northern China. The genome sequence of this strain reported here may provide insights into genomic characteristics regarding the biodegradation of organosulfur and organic nitrogen compounds under elevated temperature.

## ANNOUNCEMENT

Aeribacillus pallidus, formerly classified as Geobacillus pallidus, was proposed to be classified in a novel genus, Aeribacillus (1). Many strains of *Aeribacillus* have been isolated from sewage, oil-contaminated soil (2), high-temperature oilfields (3), hot springs (4), and a deep geothermal reservoir (5). Aeribacillus pallidus strain W-12 was isolated from Dagang oil reservoir sewage in northern China by using liquid paraffin-added Luria-Bertani (LB) medium at 60°C. Analysis of the 16S rRNA gene sequence, with 99% identity with Aeribacillus pallidus GS3372 (5), indicated that W-12 is a novel strain of *A. pallidus*.

Genomic DNA of *A. pallidus* W-12 was extracted from an overnight culture in LB medium at 60°C by using a genomic DNA isolation kit (Sangon, Shanghai, China). The TruSeq DNA sample prep kit (Illumina, USA) was used to create a paired-end library (400 bp). Genome sequencing of *A. pallidus* W-12 was carried out using the Illumina HiSeq 2500 platform at Majorbio Bio-Pharm Technology Co. Ltd. (Shanghai, China). *De novo* assembly was performed by using SOAP*denovo* (version 2.04), followed by local hole filling and base correction of assembly results using GapCloser (version 1.12) (6). The reads were quality controlled by G+C deviation and base error rate. Low-quality reads (sequencing quality value, <Q20; N content, >10%; or read length, <25 bp) were cut. The predictions of rRNA and tRNA of the genome were performed by Barrnap 0.4.2 and tRNAscan-SE version 1.3.1, respectively. Bacterial gene prediction was performed using the Glimmer 3.02 software. Functional annotation was based on BLASTp with the KEGG, Swiss-Prot, Clusters of Orthologous Groups (COG), and nonredundant (NR) databases.

A total of 2 × 2,670,109 raw data paired reads and 2 × 670,197,359 bases with a Q20 value of 97.16% were obtained from the sequencing. The final genome of strain W-12 was assembled into 140 scaffolds, the longest of which was 305,219 bp, and the *N*_50_ value was 99,755 bp. The total length of all contigs is 3,839,111 bp, and the *N*_50_ and *N*_90_ values of the contigs are 90,782 bp and 16,867 bp, respectively. Gene predictions and annotations were performed as previously described (7). The number of protein-encoding genes is 4,329, and the total genome size is 3,172,089 bp. The G+C content is 38.86%, which is close to the G+C content of *A. pallidus* type strains DSM 3670 and 8m3 (8), and the coding sequence (CDS)/genome percentage (coding percentage) was 82.6%.

Three putative alkanesulfonate monooxygenase genes, 10 putative alcohol dehydrogenase genes, 6 nitroreductase genes, and 1 2-nitropropane dioxygenase gene were identified in the genome of W-12. They were hypothesized to be related to the pathways for biological desulfurization and denitrification. Alkanesulfonate monooxygenase genes are responsible for the degradation of organosulfur compounds (9), and the 2-nitropropane dioxygenase gene catalyzes oxidative denitrification of nitroalkanes to carbonyl compounds and nitrites (6). One 3-demethylubiquinone-9 3-methyltransferase gene was also identified in this genome, which may be responsible for the metabolism of aromatics. This strain has potential for biodesulfurization and biodenitrification, which is similar to what is found with the previously reported strain Geobacillus thermoglucosidasius W-2 (6), based on genetic annotation. Moreover, enzymes from thermophiles are active and stable under extreme temperatures, broad pH ranges, and the presence of organic solvents, which may provide many different advantages from an industrial perspective (10).

### Data availability.

The raw sequence reads have been submitted to the NCBI SRA under the accession number SRR8510162, and the whole-genome shotgun project of *A. pallidus* W-12 has been deposited at DDBJ/ENA/GenBank under the accession number QURG00000000. The version described in this paper is version QURG01000000.
